# Bionic Robot with Multifunctional Leg–Arm Mechanism for In-Orbit Assembly of Space Trusses

**DOI:** 10.3390/biomimetics9090550

**Published:** 2024-09-11

**Authors:** Yuetian Shi, Qingzhang Xu, Rui Shi, Haohang Liu, Meiyang Zhang, Xuyan Hou, Weijun Wang, Zongquan Deng

**Affiliations:** 1 Research Center of Aerospace Mechanism and Control, School of Mechatronics Engineering, Harbin Institute of Technology, Harbin 150080, China; qthshiyuetian@163.com (Y.S.); 13339465939@163.com (R.S.); liuhaohang5000@163.com (H.L.); zhangmeiyanghit@163.com (M.Z.); dengzq@hit.edu.cn (Z.D.); 2 Space Structure Mechanism Technology Laboratory, China Aerospace Science and Technology Group Co., Ltd., Shanghai 201109, China; 3 Zhengzhou Research Institute, Harbin Institute of Technology, Zhengzhou 450000, China; 4 Department of Mechanical Engineering, Faculty of Engineering, The Hong Kong Polytechnic University, Hung Hom, Kowloon, Hong Kong SAR 999077, China; james_xu2003@163.com; 5 Songjiang Laboratory, Harbin Institute of Technology, Harbin 150080, China; 6 Shanghai Institute of Aerospace System Engineering, Shanghai 201109, China

**Keywords:** in-orbit assembly of space truss structures, Dynastes Hercules beetle tarsus, bionic legged robot, overall–local dynamic model

## Abstract

This article aims to address the in-orbit assembly needs of truss structures in space missions by designing a robot capable of moving on trusses and manipulating parts. To enhance the stability of the robot during movement and part manipulation, inspiration was drawn from the Dynastes Hercules beetle. Building upon detailed research on the Dynastes Hercules beetle, a biomimetic structure was designed for the robot system. Based on specific task requirements, the overall plan of the robot was developed, and its kinematic and dynamic models were derived. A prototype of the robot was created, which is capable of both movement and assembly functions, including handling spherical and rod-like objects. Through a series of experiments conducted with the robot, the research results demonstrated that the proposed design can effectively achieve the intended functions.

## 1. Introduction

Truss structures are widely used in engineering projects such as spacecraft, satellites, and space stations. They are a three-dimensional structure composed of connecting rods and nodes, providing powerful support and stability. Therefore, robots that can move freely among the trusses have unique advantages. In the field of space assembly, the assembly of truss structures usually relies on a fixed pedestal form, such as a robotic arm. This is mainly because in past space missions, the use of tools such as robotic arms has been more stable and controllable to ensure the completion of complex assembly tasks in a microgravity environment. However, traditional robotic arms have certain limitations in truss assembly. They usually need to be pre-designed and installed in specific locations of the space structure, which limits their flexibility and adaptability. In contrast, freely moving robots have shown unique advantages in truss assembly. These robots can shuttle freely between the trusses, perform various tasks, including assembly, installation, and maintenance. This flexibility allows them to adapt to different structures and task requirements, bringing greater diversity and adaptability to assembly.

Senda and Matsumoto [1] studied the assembly methods for free-flying space robots assembling space trusses and enhanced these methods through reinforcement learning. The space truss design and the method of space robot assembly by the National Aeronautics and Space Administration [2] are among the earliest systems of robotic truss assembly, primarily focused on the autonomous assembly and disassembly of an 8 m diameter truss structure composed of 102 truss elements, covered with 12 panels. Liu Dongbo [3] conducted research on impedance control based on adaptive fuzzy CNF for space robot on-orbit truss assembly operations. Oegerle and Perves [4] proposed a method for space robots to assemble large space telescope truss structures. Hu Jiaxing [5] addressed the on-orbit intelligent assembly issue for modular space trusses, defined assembly information for the newly developed truss structure, established coding rules and data structures for truss assembly, and quantitatively described the differences in features, positions, and connections of different basic units. Hayashi [6] developed a training algorithm using graph embedding and reinforcement learning to generate stable assembly paths for arbitrarily combined truss structures.

In the field of on-orbit assembly, there are four classifications of robots: autonomous flying modules, self-assembling space robots, free-flying assembly robots, and attachment-based assembly robots. The research background of this paper focuses on the manufacturing and assembly of parts within the assembly object hierarchy, opting for the attachment-based assembly robot solution. From the aforementioned research, it can be seen that the study of space truss assembly primarily concentrates on the assembly of large components and the planning of the assembly process, placing greater emphasis on the systematic and efficient nature of assembly. There is relatively little research on the operation at the part level in space working environments. Moreover, in the direction of part manufacturing and assembly, the relevant project robot driving methods mostly involve free-flying robots and self-assembling space robots using mechanical arms, with less attention given to the structures widely used in space trusses. This paper’s research mainly targets the transportation and assembly of smaller parts at the connections of large components during the on-orbit assembly processes of large space stations and space solar power stations. The assembly objects are categorized based on their size hierarchy during the on-orbit assembly, and the corresponding related projects are listed in Table 1.

The research background of this paper is focused on the in-orbit assembly of space truss structures in the space environment. In addition to the need for assembling ultra-large components, there are also assembly requirements for smaller parts. These require robots to be able to move on the truss structure and carry parts for simple assembly operations. When performing in-orbit assembly of part-level space truss structures in the space environment, there are more detailed requirements that the robots need to meet:

(1) Multi-joint: Due to the need to move and assemble parts on the truss structure, the robot must have substantial flexibility and adaptability to navigate through complex structures.
(2) Adhesion: Considering the low-gravity characteristics of the space environment, the robot must have strong adhesion capabilities to move more stably, using suction cups, claws, or other attachment devices to fix itself onto structures to achieve stable movement and operations.
(3) Modular design: To adapt to different assembly tasks, the robot can utilize a modular design, allowing flexible combination and adjustment according to task requirements, thereby achieving multifunctional operations. Legged robots have multi-joint characteristics and considerable flexibility.

Additionally, each leg of a legged robot has the same structure, meeting the requirements of modular design. Therefore, legged robots are chosen as the mode of movement for space robots, and adhesion-enhancing methods are adopted to increase movement stability.

Legged robots have always been a research hotspot since they can meet the pressing needs of some specific scenarios. Various types of legged robots, such as humanoid robots [7], quadruped robots [8], hexapod robots [9], and wheel–leg integrated robots [10], are all attempting to complete more complex tasks. For humanoid robots, the biggest challenge is how to maintain balance when carrying an object due to the lack of sufficient support points. Atkeson [11] and others have proposed a solution to this problem. For quadruped robots [12,13], if they want to carry objects, they must install additional mechanical arms for grasping because quadruped robots cannot maintain balanced movement using two or three legs. Compared with the previous two, hexapod robots are more practical and flexible in complex environments when considering balance ability and multifunctionality. This is determined by the larger support polygon and more redundant degrees of freedom that hexapod robots have.

The earliest idea for using a hexapod robot to move objects is still to add additional arms to the robot, with clear division of labor between the arms and the robot legs. Kalouche [14] developed a multi-mode walking robot called Snake Monster, which can easily adapt to configurations of hexapod, quadruped, and bipedal animals, and equipped the robot with tools for grabbing objects according to the tasks it performs. Santos [15] designed a hexapod robot for mine clearance, with a five-degree-of-freedom mechanical arm placed in front of the robot, which simultaneously handles the task of scanning the terrain. Inspired by ants in the biological world, Lewinger [16] created a small hexapod robot named Bill-Antp, which represents an early study of robots using ants as a bioinspiration object. In this research direction, we have also created a prototype that carries an additional robotic arm. However, the biggest problem with this solution is the high weight of the robotic arm, which even exceeds that of the ground-based legged robots. From both the aerospace lightweight principle and the perspective of energy conservation in robots, this solution is highly inefficient. Therefore, we are considering other alternatives.

Inheriting previous research ideas, Koyachi [17,18] and others presented a new concept for the integration of locomotion and manipulation, introducing the “integrated limb mechanism” that consists of a six-bar linkage mechanism with four degrees of freedom. This mechanism combines the advantages of legs and arms and underwent kinematic analysis. This may be one of the earliest attempts to integrate legs and arms into a robot. Later, they developed two prototype robots, MELMANTIS-1 and MELMANTIS-2, aiming to demonstrate that the prototypes could lift bottles. This four-degree-of-freedom linkage mechanism can be seen as a single-leg prototype verification, which holds significant importance for future leg–arm combined robots.

Takubo [19] from Osaka University in Japan developed the hexapod robot Asterisk, which can use two legs to grasp objects while the remaining legs are used to support the body. Bartish [20] and colleagues developed the praying mantis robot, aiming for it to carry and manipulate external objects like a praying mantis, but issues such as gait planning and balance control need to be addressed. Heppner [21,22] proposed the hexapod robot LAURON V for planetary exploration. It grabs objects with gripping tools directly mounted on the robot’s legs, stores them on the robot’s back, and achieves a five-leg gait in this process. Yi Zheng [23] introduced a hexapod robot with two integrated leg–arm limbs, capable of gripping and cutting operations, and analyzed the gait switching problem under single-limb and dual-limb operation. Hua Deng [24] presented a similar concept, proposing several methods to convert one or two legs of a hexapod robot into arms, using the other legs to handle the movement of carrying objects. They conducted more detailed experiments and demonstrated the effectiveness of the carrying method by having the robot carry bottles and boxes.

To improve the precision and efficiency of legged robots, recent research has been focused on two main directions. The first direction is enhancing the perception accuracy of legged robots. Yao Chen [25] designed a terrain-adaptive foot sensor for legged robots walking on soft slopes. Shi Guowei [26] installed sensors on robots to perceive 6D contact forces on terrains of varying stiffness and estimate the local inclination of contact surfaces; the proposed vision-based sensory foot can enhance the contact perception capability of legged robots in different environments. Yaodong Wang [27] developed a heuristic contact position estimator inspired by symmetrical deformation characteristics, with sensors used in an array format. Zhaoyang [28] Wang proposed a triboelectric tactile sensor for a legged magnetic adhesion climbing robot, aimed at detecting ship hull plates.

The second direction is improving the control algorithms of robots. Shahbazy [29] proposed a new foot placement planning method based on the A* algorithm and kinematic manipulability. Safeer Ullah [30] presented a novel design for a distributed fixed-time synchronization controller based on neuro-adaptive non-singular terminal sliding mode control for higher-order multi-agent nonlinear systems. Xinxing Tang [31] proposed an improved Artificial Potential Field (IAPF) method to enhance path planning efficiency and obstacle avoidance capability. Junfeng Xue [32] combined the Global Optimal Path Search Tree (GOPST) algorithm with a prior path estimation method using Graph Convolutional Networks (GCNs) to optimize obstacle avoidance for robots.

From the above research, it can be seen that the multifunctional legged robot solution with both leg and arm functions is feasible. If the attachment-based assembly robot solution is adopted in the direction of space part assembly, and a legged robot is chosen to perform movement and object manipulation in the unique environment of a truss, it will facilitate the future exploration and operation of more complex structures. At the same time, it will ensure the operability and continuous updating of equipment without the need to launch additional human resources from Earth.

Therefore, the research plan in this paper adopts a four-degree-of-freedom hexapod robot scheme, initially achieving the robot’s mobility on trusses and the function of simple object manipulation, thus completing the verification of the scheme’s feasibility. The specific research contents are as follows:

Firstly, we utilize a bionic approach to conduct an in-depth study of the tarsus of the Dynastes Hercules beetle. By analyzing its biological characteristics and movement mechanisms, we designed a bionic tarsal structure that can achieve maximum gripping efficiency, providing effective support points for the robot’s movement on space truss structures. This bionic tarsal structure can simulate the natural movement of the beetle, significantly enhancing the robot’s stability and flexibility in complex environments.

Secondly, we propose a combined grasping and advancing scheme. This approach designs a bionic structure at the third joint to provide support points on the truss for the robot, and uses the fourth joint to grip the truss tightly to provide sufficient friction for forward movement. This allows the robot to achieve stable movement on the truss structure while maintaining sufficient gripping force without affecting other tasks.

Finally, compared to traditional schemes that equip robots with mechanical arms, we adopt a leg–arm reuse scheme and design a 4 + 2 working gait. By fabricating a robot prototype, we verified the feasibility of this design. Additionally, we adopted a variable body configuration to increase the working space for handling components, providing technical assurance for subsequent assembly tasks. This innovative design not only simplifies the robot’s structure but also enhances its working efficiency and operational range.

## 2. Overall Robot Scheme and Bionic Applications

**Figure 1:**
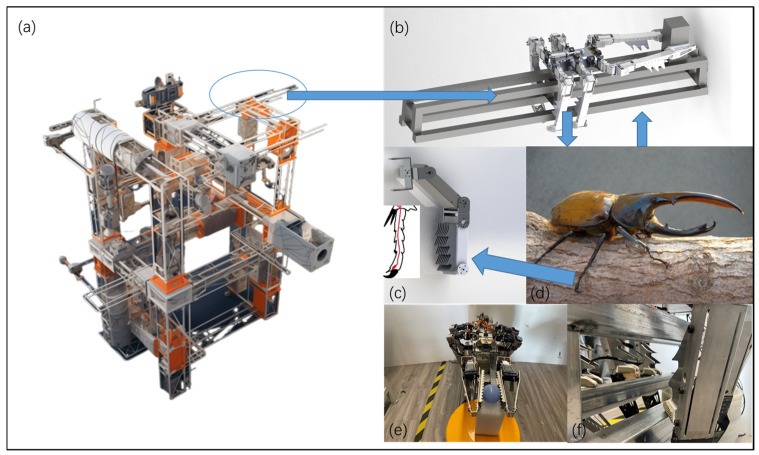
Schematic diagram of the overall design of the truss assembly biomimetic robot on orbit. (**a**) Schematic diagram of the space truss assembly work environment. (**b**) Schematic diagram of robot operation. (**c**) Biomimetic tarsal joint structure. (**d**) Biomimetic object Dynastes Hercules. (**e**) Biomimetic structure for assembly function. (**f**) Biomimetic structure for mobility function.

The research background of this study is focused on the in-orbit assembly of space truss structures, requiring robots to move on the truss structure and be able to move parts for simple assembly operations. Compared to free-floating robots and robotic arm robots, attachment-based legged robots have unique advantages in the in-orbit assembly of space truss structures. As they can attach to the truss structure, attachment-based robots can achieve more precise positioning and movement, aiding in the accurate placement and assembly of components. Compared to free-floating robots that require thrusters for movement, attachment-based robots operating in a microgravity environment benefit from the support of the truss structure, resulting in more energy-efficient movement. In comparison to robotic arm robots, attachment-based robots can move in multiple directions along the truss structure, possessing higher flexibility and suitability for operations in complex space environments. Figure 1a shows a schematic diagram of the space truss structure.

The in-orbit assembly of space truss structures requires robots to be able to move, position, and assemble parts in complex environments, necessitating good adhesion and stability to ensure efficient and accurate operations. Many organisms in nature exhibit excellent locomotion and adhesion capabilities in different environments. For example, the claws and foot structures of insects can provide valuable inspiration for robot design. Figure 1d illustrates the crawling diagram of the Dynastes Hercules beetle in nature. The Dynastes Hercules beetle has a unique foot structure, with special morphological and microstructural features on its feet that give it excellent adhesion performance on smooth and irregular surfaces. Its foot features include elongated antennae, foot pads, and claws adapted to different surfaces, making it a valuable bioinspiration model worth studying.

In previous studies, the tarsal segment structure of the Dynastes Hercules beetle was used as a reference to design a foot attachment mechanism for climbing between the truss bars [33], as shown in Figure 1c. The research contents of previous work are shown in Table 2.

Compared to other insects with adhesion capabilities, the Dynastes Hercules beetle has its unique advantages. Among the world’s strongest animals, the Dynastes Hercules beetle can lift 840 times its own body weight, ranking first. In contrast, leafcutter ants and gorillas can lift 50 times and 10 times their body weight, respectively. This demonstrates that the Dynastes Hercules beetle has astonishing strength and lifting ability in the animal kingdom, far surpassing other animals in this regard. During biological observations, the Dynastes Hercules beetle can climb a tree trunk and support the weight of another beetle solely with its tarsal structures. During the research process, high-magnification microscopy was used to observe the structure of the Dynastes Hercules beetle, and three-dimensional model scanning and reproduction were conducted. Ultimately, the research focused on the tarsal structures of the beetle. In this process, a theoretical model of the tarsal attachment process was established, identifying several factors that affect hooking efficiency, and experimental tests were conducted on the tarsal structure. Tests were performed on six different surface parameters of materials and four different contact methods. Additionally, simulation software was used to analyze the contact process between 32 different sizes of biomimetic tarsal structures and truss bars. The above research provided the prerequisites for the robot’s ability to crawl on the truss in this study.

The bionic legged robot used for in-orbit assembly of trusses is shown in Figure 1b, with a rectangular layout for the overall body platform. The main body of the robot is made of hollowed aluminum alloy processed parts, ensuring high strength while reducing the robot’s mass. Six robot legs are symmetrically distributed on both sides of the robot, with three legs on each side, aligning well with the shape of the truss and facilitating the robot’s movement on the truss.

**Figure 2:**
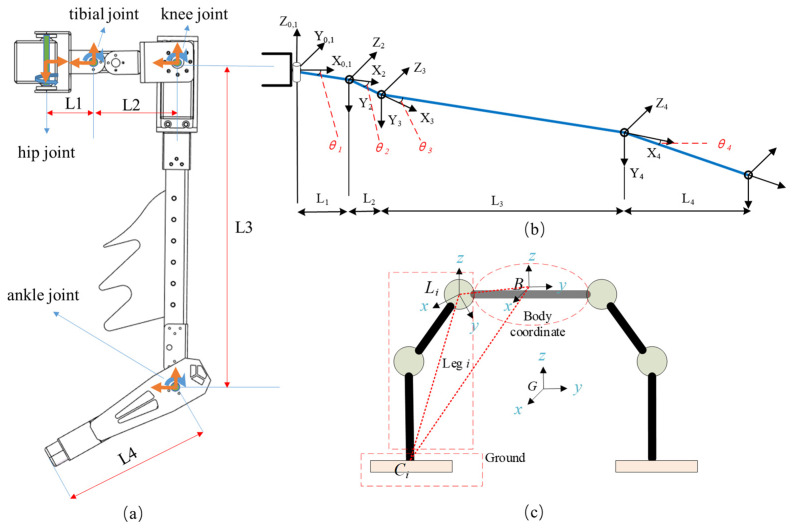
Kinematic structure and coordinate system schematic. (**a**) Joint distribution and variable annotation of a single robot leg. (**b**) Leg DH coordinate system. (**c**) Establishment of the coordinate system for the entire robot.

The six robot legs of the bionic legged robot have identical structural layouts, each leg consisting of four actively rotating degrees of freedom in series. The joint closest to the body is the hip joint (designated as Joint 1), with its axis perpendicular to the plane of the body platform. Connected to the hip joint is the tibial joint (designated as Joint 2), aligned similarly to Joint 1. The axes of the knee joint (Joint 3) and ankle joint (Joint 4) are perpendicular to the axis of Joint 1. The robot body platform has a rotating degree of freedom that can be raised while performing assembly tasks between two robot legs to gain more workspace. The rods of Joints 3 and 4 are left with longer lengths as positions for placing bionic structures. Figure 2a shows the distribution of each joint.

The functionalities designed for the robot in this paper are divided into two main categories: mobility (crawling on trusses) and assembly (picking up and placing parts). In the mobility function, each robot leg has three degrees of freedom and uses a designed biomimetic mechanism to attach to truss bars, allowing it to move like a hexapod robot, or alternatively, for higher stability, adopt a posture for pole-climbing. The biomimetic mechanism is installed at the third joint of the robot leg, as shown in Figure 1f, serving as the attachment point for the mobility function. Due to the difference in the diameter or cross-sectional shape of the truss rod, the difference in the lateral distance between adjacent rows of hook claws can allow the robot to better adapt to different hooking conditions, thereby improving the robot’s hooking success rate and stability.

For example, when dealing with truss rods with a large diameter variation, if the lateral distance difference between adjacent rows of hook claws is the same, some hook claws may not be able to fully grip the rod, affecting the hooking effect. However, if the lateral distance difference between adjacent rows of hook claws is different, it can be optimized according to the change in rod diameter, allowing the hook claws to grip the rod better and achieve stable hooking.

In the assembly function, the two legs on the head side act as the operating mechanism, while the other four legs remain stationary. The legs serving as the operating mechanism have four degrees of freedom, and in coordination with the rotational freedom of the body platform, they use the end effectors of two robot legs to grasp and manipulate parts, thus achieving the assembly function. To enhance gripping capability, as shown in Figure 1e, bionic mechanism is installed at the fourth joint of the assembly leg.

## 3. Theoretical Models Related to Robots

The difference between the robot studied in this paper and traditional hexapod robots is that the support point for movement on trusses is at the third joint, rather than at the end of the robot’s leg. The robot’s legs use three degrees of freedom for crawling and four degrees of freedom for moving objects. To extend the manipulation range for moving objects, an additional degree of freedom was added to the body. Therefore, a modular analysis of the robot’s whole-body kinematics was conducted considering multifunctionality. Through kinematic modeling, we can determine the motion trajectory of each joint when the robot performs specific actions, enabling precise control and path planning. The robot legs are categorized into three types: swinging legs without support points, support legs using the third joint for support, and assembly legs using the fourth joint for assembly functions. Dynamic analysis is performed according to different working conditions, considering the impact of external forces on the robot, as well as internal factors such as inertia and friction affecting the robot’s movement. Through dynamic modeling, we can understand the forces and torques the robot experiences during movement and how these forces and torques affect the robot’s acceleration and speed changes.

### 3.1. Kinematics Analysis

Analyzing the kinematics of robots can provide kinematic theories for mobility and assembly functions. Here, we only list the kinematic model of the more complex four-degree-of-freedom assembly function [34]. It can be inferred from the forward kinematics end-effector transformation matrix:

(1) $$\begin{array}{l} p_{x}=c_{1}\left(L_{4}c_{234}+L_{3}c_{23}+L_{2}c_{2}+L_{1}\right)\\ p_{y}=s_{1}\left(L_{4}c_{234}+L_{3}c_{23}+L_{2}c_{2}+L_{1}\right)\\ p_{z}=L_{4}s_{234}+L_{3}s_{23}+L_{2}s_{2} \end{array}$$

where $p_{x},p_{x},p_{x}$ are the three-dimensional coordinates in the contact transformation matrix, $L_{i}$ is the length of the link corresponding to joint *I*, $\theta_{i}$ is the rotation angle of joint *I*, and $s_{i}$ and $c_{i}$ are the sine value and cosine value of the rotation angle of joint *i*.

By using analytical methods to solve the inverse kinematics of a single leg, the inverse expression of the four joint angles can be obtained

(2) $$\begin{array}{l} \theta_{1}=\arctan(p_{y}/p_{x})\\ \theta_{2}=\arctan\left(p_{z}/c-L_{1}\right)\\ \theta_{3}=\arctan\left(\left(p_{z}-L_{2}s_{2}\right)\left(L_{4}s_{4}+L_{3}\right)-\left(2\sqrt{{p_{x}}^{2}+{p_{y}}^{2}}\left(L_{1}+L_{2}c_{2}\right)\right)L_{4}s_{4}\right)\\ \theta_{4}=\arctan\left(\frac{\pm \sqrt{1-A^{2}}}{A}\right) \end{array}$$

where

(3) $$\begin{array}{l} c=\sqrt{{p_{x}}^{2}+{p_{y}}^{2}}\\ A=\left(\begin{array}{l} {p_{x}}^{2}+{p_{y}}^{2}+{p_{z}}^{2}+L_{1}^{2}+L_{2}^{2}-L_{3}^{2}-L_{4}^{2}\\ +2L_{2}\left(L_{1}c_{2}-p_{z}s_{2}\right)\pm 2\sqrt{{p_{x}}^{2}+{p_{y}}^{2}}\left(L_{1}+L_{2}c_{2}\right) \end{array}\right)/2L_{3}L_{4} \end{array}$$

After conducting a kinematic analysis of a single robot leg, it is necessary to further perform a kinematic analysis of the entire robot. Whether using the third joint for support during movement or the fourth joint for grasping and assembly, a stable closed-loop support chain needs to be formed. Therefore, a coordinate system for the entire robot is established, considering the number of joints as a variable, as shown in Figure 2b.

Use {P} (physical) to represent the body coordinate system of the hexapod robot and {G} (ground) to represent the global coordinate system. In this paper, the ground coordinate system does not only represent the ground but also represents a more general fixed coordinate system that maintains contact with the robot. Establish the coordinate system {L} at the connection between the robot leg and the body, where $L_{i-x_{Li}y_{Li}z_{Li}}$ represents the coordinate system {Li} (leg) of the *i*-th robot leg. The point Li coincides with the connection of the *i*-th robot leg and the body, point B (body) is the coordinate origin of the body coordinate system, and point *C_i_* is the contact position of the robot leg with the global coordinates, which can be described by roll, pitch, and yaw angles for the posture of the body.

The expression of the foot contact point in the coordinate system {Li} can be obtained.

(4) CiLi=R−1LiP(R−1GP(CiG−BG)−LiP) i=1~n

Given the parameters of various positions in the known global coordinate system, obtaining the expression of $C_{i}$ in coordinate system {Li} can be used in the kinematic calculations of a robot’s single leg. It can be seen that the fixed value of point Li in the body coordinate system {P} can be used to represent the foot contact point in the robot leg coordinate system {Li} by Ci point and B point in the global coordinate system {G}. Furthermore, the kinematics of a single leg can derive the functional relationship between the reference point B of the body in the global coordinate system and the angles of each driven joint of each robot leg. In analyzing the velocity of the entire system, the relationship between the position vectors of the robot’s support leg joints in the global coordinate system {G} and the generalized velocity vector of the body is determined. The relationship between the generalized velocity of the body, denoted as VBG, and the input speed of the *i*-th leg, denoted as θ.pLi, can be obtained.

(5) RPG RLiP·JviLi·θ.pLi=−x˙By˙Bz˙B−ωyB(Ciz−Bz)−ωzB(Ciy−By)ωzB(Cix−Bx)−ωxB(Ciz−Bz)ωxB(Ciy−By)−ωyB(Cix−Bx)=f(VBG)

For ease of representation in subsequent expressions, the mapping matrix is expressed as a product of multiple matrices.

(6) −x˙By˙Bz˙B−ωyB(Ciz−Bz)−ωzB(Ciy−By)ωzB(Cix−Bx)−ωxB(Ciz−Bz)ωxB(Ciy−By)−ωyB(Cix−Bx)=−I3×3Ki·VBG

In this context, $K_{i}$ is the matrix related to the support point C_i_ of the *i*-th support leg.

The mapping matrix between the generalized body velocity and the input speed of the *i*-th leg is:

(7) diagRPG·LiPR·JviLi.θ.pL1θ.pL2⋮θ.pLi=−I3×3K1−I3×3K2⋮⋮−I3×3Ki.VBG

Based on the velocity relationship equation, the acceleration relationship is solved. By organizing the terms related to the generalized acceleration of point B, we can further obtain

(8) RPGRLiP·aCiLi=−I3×3Ki·APG−ωB×(GvCi−vBG)−ωB×(PGRvCiP)

Therefore, establishing the equation in the form of the above equation for each support leg, we can obtain the mapping relationship matrix between the generalized body acceleration and the acceleration of the foot point *C_i_* in the robot coordinate system {Li} when the *i*-th leg is the support leg.

(9) diagRPG·LiPR·aC1L1aC2L2⋮aCiLi=−I3×3K1−I3×3K2⋮⋮−I3×3Ki.ABG−RPG·D1RPG·D2⋮RPG·Di.ωB−RPG·E1RPG·E2⋮RPG·Ei.ωB

where *D_i_* is the anti-symmetric matrix of RPG·LiPR·JviLi·θ.pLi, and *E_i_* is the anti-symmetric matrix of RLiP·JviLi·θ.pLi.

### 3.2. Robotic Dynamics Analysis

During the process of constructing the overall dynamic model of the robot, the research subject in this paper has different degrees of freedom distributions and component configurations. All constraints are considered to involve a significant amount of complex calculations. Therefore, an overall–local analysis method is adopted. The robot leg is analyzed as a whole to determine its dynamic situation in different states. The generalized active forces and generalized inertia forces in different coordinate systems are calculated, and finally, the Kane method is used for the overall dynamic modeling of the robot.

The research object of the dynamic analysis of the entire robot system is divided into four parts: the body, the swing legs moving relative to the body, the support legs stationary relative to the body, and the assembly legs moving relative to the body.

Due to space limitations, this paper focuses on the dynamic analysis of the swinging leg, which has a more complex working condition. The differences between the supporting leg and the assembly leg will be listed separately.

As mentioned in the previous section, the generalized velocity VPG and the generalized acceleration APG of the body are in the global coordinate system {G}. In the robot system of this paper, the number of swing legs is denoted as m (0≤m≤6), the position vector is denoted as CmsG of the robot end contact point $C_{m}$ in the global coordinate system, and the position vector is denoted as CmsLm in the robot foot coordinate system $\left\{L_{m}\right\}$. The relationship between the two is:

(10) CmsG=BG+RPG·LmPR·LmCms

Taking the derivative and applying the angular velocity vector, we can obtain

(11) vCmsG=vBG+ωBG×PGR·LmPR·LmCms+PGR·ωBLm×LmPR·LmCms+PGR·LmPR·LmvCms

Continuing with the derivative, considering the Coriolis acceleration, we can obtain

(12) αCmsG=GαB+GαB×RPG·LmPR·LmCms+2·GωB×RPG·LmPR·LmvCms+PGR·LmPR·LmαCms

The motion of the swing leg is divided into two sub-motions for analysis:

Case 1: The sub-motion where the robot foot follows the body movement. In this case, the robot foot is in a relatively stationary state relative to the body, and the velocity vector VXαG of the centroid X point of any component of the *m*-th robot foot in the global coordinate system is:

(13) VXαG=vXαGωXαG=vBG+GωB×RPG·LmRP·LmXmsωBGωB=IE10IGVB

where $E_{1}$ represents the anti-symmetric matrix of RPG·LmRP·LmXms. Taking the first derivative of both ends of the above equation with respect to time t, the acceleration of the centroid point X can be obtained.

(14) AXαG=αXαGεXαG=αBG×RPG·LmPR·LmCms+2·GωB×RPG·LmPR·LmvCms+PGR·LmPR·LmαCmsεB

The expression for the inertial force and inertia moment of the rod with the centroid at X in {G} is:

(15) fXmαG=−mX·GaXα nXmαG=−GIXm·GεXα −GωXα ×IXmG·GωXα 

Case 2: The relative sub-motion between the robot foot and the body. In this case, the origin of the first link of the robot foot is the point Li of the coordinate system. The velocity and acceleration of the centroid X point of any component of the *m*-th robot foot in the coordinate system $\left\{L_{i}\right\}$ are the same as the velocity and acceleration in the previous section’s single-leg kinematics. The velocity vector in the global coordinate system {G} is

(16) ωpsmG=PGR·LmPR·Lmωpsm=PGR·LmPR·J−1·LmvCmsVYβG=vYβGωYβG=RPG·LmPRRPG·LmPR·JvCmsLm=E2·JvCmsLm

where J is the full Jacobian matrix from the actuator to the end point velocity of the foot. By taking the first derivative of both sides of the above equation with respect to time t, the acceleration vector of the centroid X in {G} can be obtained.

(17) AXβG=aXβGεXβG=E2·J·LmvCms+E2·J.·LmvCms+E2·J·LmaCms

The expression of the inertial force and the inertial force moment for the link with the centroid at X in {G} is:

(18) fXmβG=−mXg+GaXβnXmβG=−GIXm·GεXβ−GωXβ×IXmG·GωXβ

When the *m*-th mechanical leg is in a swing phase, its driving force in {G} is

(19) FqsmG=PGR·LmPR·Lmτd1smτd2sm…τdrsmT

where FqsmG is the driving force of the *m*-th swinging leg in the ground coordinate system and $\tau_{di}^{sm}$ is the driving force of the *i*-th joint of the *m*-th swinging leg.

Then, apply Kane’s virtual work principle to solve the relationship between the generalized active force and the relative velocity. When the robot foot is in the swing phase, the active force expression for any r components of the swing leg is as follows, representing the set of active forces ($F_{X1},\;F_{X2},\;\cdots F_{Xr}$) for r components.

(20) FX=GFqsm+GFXmβ=PGR·LmPR·τd1smτd2sm…τdrsmT+fXmβGnXmβG=PGR·LmPR·τd1smτd2sm…τdrsmT+∑r=1n−mXr·GaXrα −GIXrm·GεXrβ−GωXrβ×IXrmG·GωXrβ

The expression of the inertial force for any *r*-th component of the swing leg is FXrmβ*G because the relative motion of the swing leg to the body does not exert a driving action on the mechanical leg.

(21) FXmr*G=GFXrmβ*=fXrmβGnXrmβG=−mXrg+GaXrβ−GIXrm·GεXrβ−GωXrβ×IXrmG·GωXrβ

The detailed analysis of the swing leg was presented in the previous text, including the analysis of velocity and acceleration, as well as dynamic analysis. Here, we will not go into detailed descriptions of the theoretical derivation for the support leg and task leg. It is worth noting that when the robot foot is in the support phase, the expression of active force should take external factors into consideration; thus, the expression of active force for the *r*-th component of m robot feet is:

(22) $$F_{Z}^{mr}=\left(\begin{array}{c} F_{Wm}\times (r_{mr}-r_{mci})+\tau_{dr}^{zm}\cdot q_{mr}\\ F_{Wm}+m_{mr}g \end{array}\right)$$

where $F_{Wm}$ is the external force of the *m*-th robot foot; for the *r*-th component of the *m*-th foot, $r_{mr}$ is its position vector, $\tau_{dr}^{zm}$ is its joint driving force, and $q_{mr}$ is the direction vector on it; $r_{mci}$ is the position vector of the end point of the *m*-th foot.

When the robot foot is in the support phase, the inertial force expression of the *r*-th component of the *m*-th robot foot is as follows:

(23) FZmr*G=fZmrGnZmrG=−mmrg+GaZmr−GIZmr·GεZmr−GωZmr×IZmrG·GωZmr

When the robot foot is used for assembly function, the inertial force expression of the *r*-th component of the *m*-th robot foot is as follows:

(24) FYmr*G=fYmrGnYmrG=−mmrg+GaYmr−GIYmr·GεYmr−GωYmr×IYmrG·GωYmr

The body is connected to six joints, and its active force and inertial force can be represented more intuitively in another way. The active force of the body can be represented as:

(25) $$\left(\begin{array}{c} F_{B}\\ M_{B} \end{array}\right)=\left(\begin{array}{c} m_{B}g+F_{W}\\ -\sum_{i=1}^{6}\tau_{i1}\cdot {\vec{r}}_{i1} \end{array}\right)$$

where $\tau_{i1}$ is the driving force of the first joint of the *i*-th robot foot, and ${\vec{r}}_{i1}$ is the direction vector of the first joint of the *i*-th robot foot.

The inertial force of the body can be represented as:

(26) $$\begin{array}{l} \left(\begin{array}{c} F_{B}^{*}\\ M_{B}^{*} \end{array}\right)=\sum_{j=1}^{6}\left(\left(\begin{array}{c} I_{B}\left(\begin{array}{c} J_{1j}^{B}\\ J_{2j}^{B}\\ J_{3j}^{B} \end{array}\right)\\ 0 \end{array}\right)+m_{B}\left(\begin{array}{c} 0\\ \left(\begin{array}{c} J_{4j}^{B}\\ J_{5j}^{B}\\ J_{6j}^{B} \end{array}\right)+\left(\begin{array}{c} J_{1j}^{B}\\ J_{2j}^{B}\\ J_{3j}^{B} \end{array}\right)\times r_{cB} \end{array}\right)\right){\overset{¨}{q}}_{B}\\ +m_{B}\left(\begin{array}{c} 0\\ \omega_{B}\times \left(\omega_{B}\times r_{cB}\right) \end{array}\right) \end{array}$$

where $I_{B}$ is the inertia moment of the body, $J^{B}$ is the Jacobian matrix corresponding to the body, and $r_{cB}$ is the position vector of the centroid point B of the body. For a system composed of n particles, the partial velocity of the *i*-th particle with respect to the *j*-th degree of freedom is defined as:

(27) $$P_{i}v_{ij}=\frac{\partial {\dot{r}}_{i}}{\partial q_{j}}$$

The generalized active force of an n-degree-of-freedom robot with respect to the *j*-th degree of freedom is equal to the sum of the product of the active force of each degree of freedom and the partial velocity of the corresponding degree of freedom.

(28) $$K_{j}=\sum_{i=1}^{n}F_{i}\cdot P_{i}v_{ij}=\sum_{i=1}^{n}F_{i}\cdot \frac{\partial {\dot{r}}_{i}}{\partial q_{j}}$$

Similarly, the generalized inertial force of the *j*-th degree of freedom is equal to the sum of the product of the inertial force of each degree of freedom and the partial velocity of the corresponding degree of freedom

(29) $$K_{j}^{*}=\sum_{i=1}^{n}F_{i}^{*}\cdot P_{i}v_{ij}=\sum_{i=1}^{n}F_{i}^{*}\cdot \frac{\partial {\dot{r}}_{i}}{\partial q_{j}}$$

The dynamic equation of the robot is as follows, where n is the number of degrees of freedom of the robot.

(30) $$\left\{\begin{array}{c} K_{1}+K_{1}^{*}=0\\ K_{2}+K_{2}^{*}=0\\ \cdots \\ K_{n}+K_{n}^{*}=0 \end{array}\right.$$

The dynamic model describes the relationship between the motion of the controlled object and the input forces or torques of the drive units. By establishing the model, the obtained curves indicate which joint input has a greater impact. Through the dynamic model, the robot’s design parameters are optimized to achieve the goal of optimized design.

## 4. Experiments

The previous section introduced the robot’s mobility and assembly functions. This section will verify the corresponding functions through experiments.

The experimental platform is a constructed simulation of a space truss structure, including standard metal rods and connectors to mimic a real in-orbit working environment. The robot system uses a bionic six-legged robot, which is equipped with a bionic foot attachment mechanism and high-degree-of-freedom joints. The robot body is made of aluminum alloy to ensure it is lightweight yet strong enough. The communication system uses wireless communication modules to achieve communication between the upper and lower computers. The lower computer’s chip is the Broadcom BCM2711 (Broadcom Inc., San Jose, CA, USA), a quad-core Cortex-A72 (ARM v8) 64-bit processor.

In the initialization state, the six-legged robot is placed at the starting position of the experimental platform, ensuring that all joints and sensors are in normal working condition.

### 4.1. Analysis of Body Joint Functions

When the robot performs assembly functions, it uses the four degrees of freedom of the front two feet to carry out the assembly tasks, while the other four feet provide movement and support functions. At the same time, to increase the movement range of the object to be gripped and to provide greater driving force for manipulating larger objects, a DC motor was added as an auxiliary drive in the middle position of the body near the assembly legs. The main dimensional parameters of the robot are shown in Table 3.

Using random functions in the MATLAB 2021 to generate random variable values for robot joint angles, by substituting the obtained random variable values into the forward kinematics equation, the position coordinates of the robot end effector can be obtained. By writing a program and looping the code N times, you can obtain N random variable values for each joint of the robot and the position coordinates of the end effector. Using the three-dimensional scatter command to plot the positions can visually represent them in three-dimensional coordinate space, providing a more intuitive image of the robot’s workspace. The range of motion of the robot joints is shown in Table 4.

**Figure 3:**
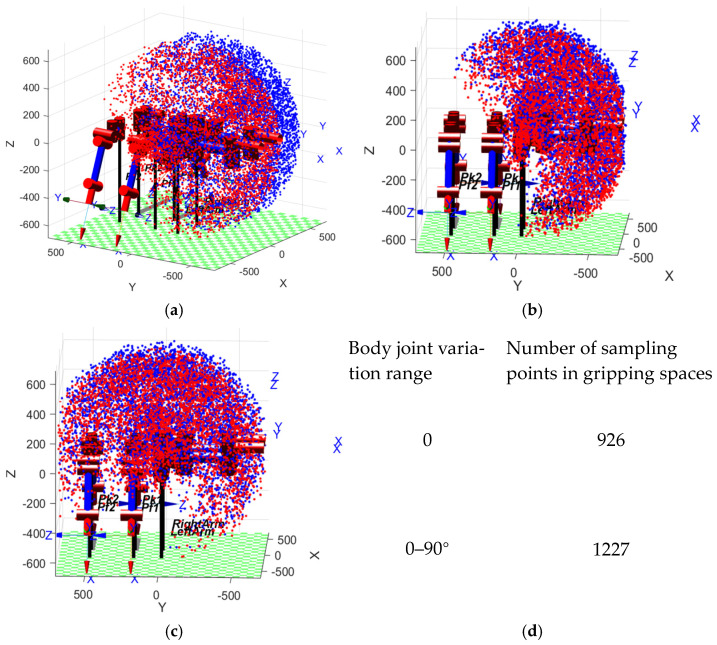
MATLAB assembly workspace analysis. (**a**) Primary perspective of the workspace; (**b**) no body joint YOZ perspective; (**c**) body joint YOZ perspective; (**d**) data of gripping space for assembly functions.

The Figure 3 shown above is the analysis of the workspace of the front two feet in MATLAB. The analysis focuses on the gripping space for assembly functions, so the workspaces of the two front feet are analyzed separately, and the solution at the overlapping part of the workspaces is the gripping space for assembly functions. Modeling the robot in MATLAB and displaying it with a scatter plot facilitates a more intuitive understanding.

From the Figure 3, the distribution of 5000 random points in the workspace can be seen. Taking the range of motion of the body joint controlled by the DC motor as 0–90°, comparing the YOZ perspective as shown in Figure 3c with the body joint angle fixed in Figure 3b, it is evident from the figure that the workspace volume has increased. From the comparison of Figure 3b,c, it can be seen that the gripping space for parts has significantly improved. By statistically analyzing the volume distribution data of the red and blue sampling points and calculating the common space volume data, we summed the sampling points in the common space. When the body joint angle does not change, the number of sampling points in the common space is 926. When the range of body joint angle changes from 0 to 90 degrees, the number of sampling points in the common space is 1227. This shows that the range of motion with body joints increased by 32.5% compared to without body joints.

### 4.2. Experimental Verification of Mobility Function

**Figure 4:**
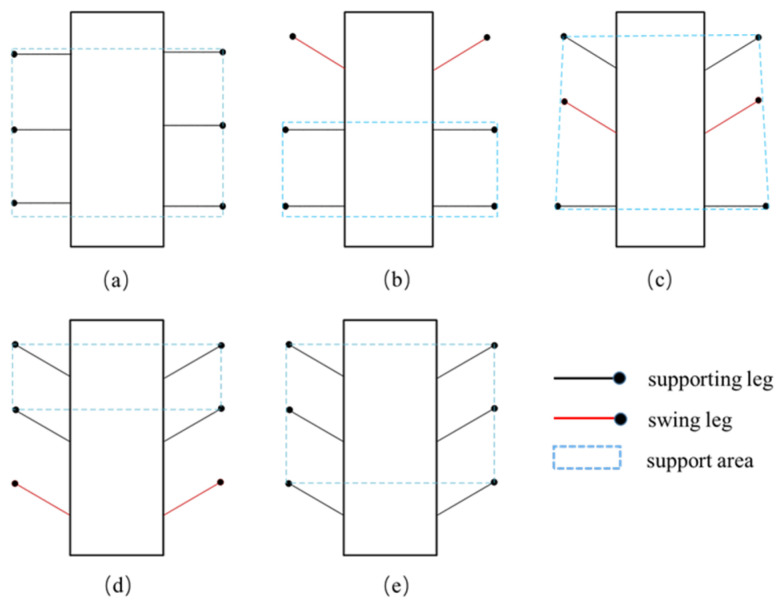
Schematic diagram of hexapod movement gait. (**a**) Initial state of the robot. (**b**) First two legs swing, last four legs support. (**c**) Middle legs swing. (**d**) Last two legs swing, first four legs support. (**e**) Hexapod supports body moving forward.

Experimental verification of the mobility function is divided into two gaits: hexapod gait movement on truss bars and quadrupedal movement gait. Taking the hexapod gait as an example, the gait is described. In Figure 4, the red represents the swinging legs, black represents the supporting legs, and the blue dashed box represents the support area.

When designing the gait of a hexapod robot suitable for climbing on trusses, factors such as stability, energy efficiency, and adaptability need to be considered. Unlike traditional hexapod robots that crawl on the ground, climbing on trusses imposes higher demands on the support points of the robot’s feet. It is essential to ensure the robot’s stability on the truss and to avoid collisions that could disrupt the balance of the robot body during movement.

The most common tripod gait of hexapod robots involves three legs leaving the ground simultaneously while the other three legs support the robot, providing high speed and good stability. However, when applying the tripod gait on trusses, due to the requirement of only three support points needing simultaneous contact with the truss, otherwise the robot is prone to tipping over, a more stable ripple gait is chosen instead. The ripple gait is a variant of the static gait where leg movements occur sequentially like waves, with one to two legs moving at a time while the rest remain in supporting positions, ensuring at least four support points are maintained.

**Figure 5:**
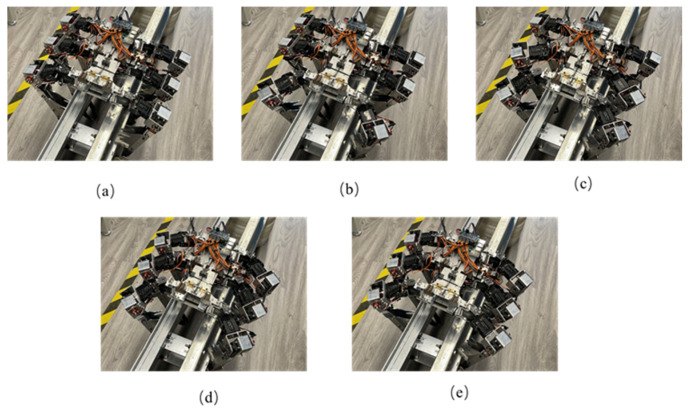
Experimental diagram of robot truss movement (hexapod posture). (**a**) Initial state of the robot. (**b**) First two legs swing, last four legs support. (**c**) Middle legs swing. (**d**) Last two legs swing, first four legs support. (**e**) Hexapod supports body moving forward.

Figure 5 shows the prototype robot achieving truss climbing in the hexapod posture, following the movement sequence depicted in the gait diagram. The swinging legs are used for positional adjustment. After all six robot legs have adjusted their positions, the hexapod supports the body moving forward. The experiment video is shown in Video S1.

**Figure 6:**
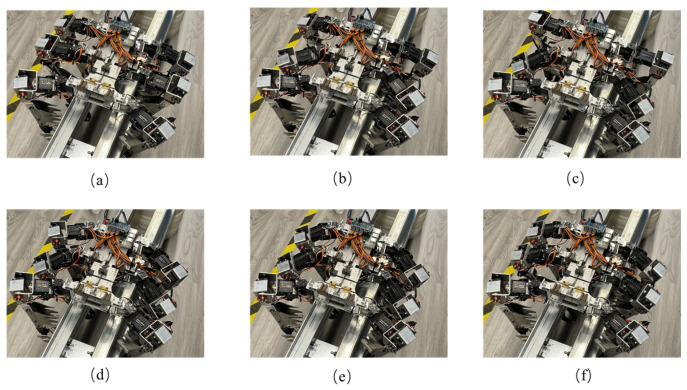
Experimental diagram of robot truss movement (quadruped posture). (**a**) Initial state of the robot. (**b**) Middle two legs swing. (**c**) Middle legs press down and grip. (**d**) Last two legs swing. (**e**) Last two legs press down and grip. (**f**) Quadruped supports body moving forward.

The robot also tested the mobility function in the quadruped posture. It is worth noting that in the quadruped posture, the robot has fewer support points. The initial design chose a static gait with single-leg movement to ensure that at least three legs support the robot in each step. However, due to the unique environment of trusses, after testing, a ripple gait was chosen with the additional method of pressing down and gripping with the robot legs to enhance stability. In Figure 6b, the middle two legs of the robot adjust their posture, with both robot legs initially positioned higher. As the robot moves to Figure 6c, the middle two legs lower their position, corresponding to the fourth degree of freedom of the robot legs executing a gripping action, which satisfies the stability requirement with both robot legs gripping. This method of movement offers faster speed and simpler control compared to a static gait. The experiment video is shown in Video S2.

### 4.3. Experimental Verification of Assembly Function

The verification of the assembly function mainly focuses on validating the feasibility of the robot in grasping, moving, and manipulating parts. For the working environment of truss structures, spherical connectors and rod-like components of the truss main structure were selected as the parts to be grasped.

**Figure 7:**
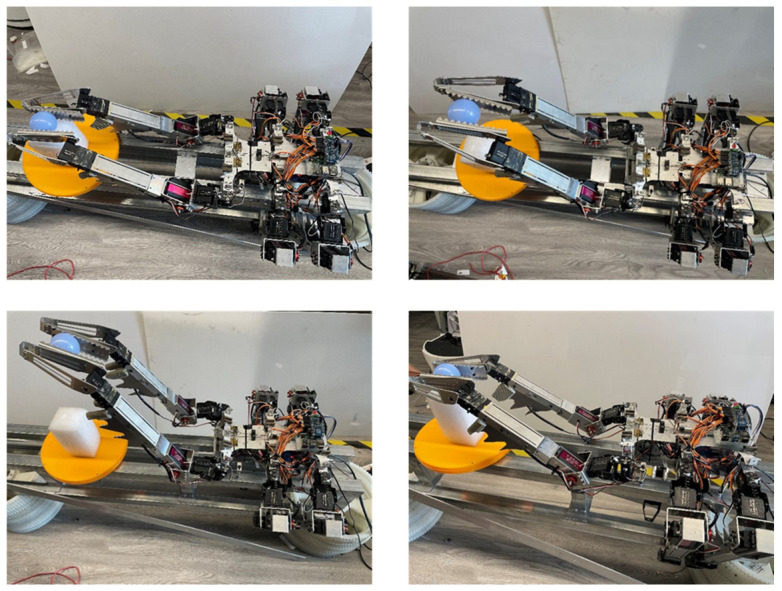
Robot gripping spherical part experiment.

**Figure 8:**
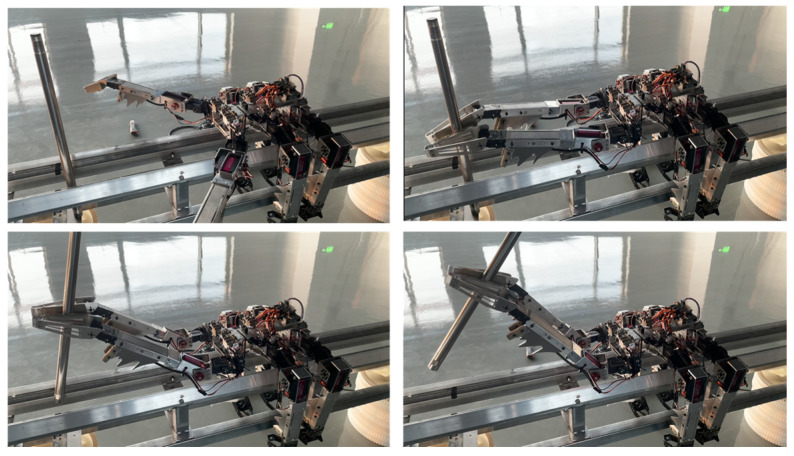
Robot gripping rod part experiment.

Figure 7 and Figure 8, respectively, show the process of the robot gripping spherical and rod parts. In the operation process of the spherical part, the robot lifts and manipulates the ball part. In addition to picking up and putting down actions, it can swing left and right, as concluded in the previous analysis. At the same time, the biomimetic structure of the fourth joint acts like a guide rail, preventing the ball part from falling during the swinging process. In the operation process of the rod part, besides performing the functions mentioned for the spherical part, due to the relatively long length of the part, lifting it can cause swinging due to lateral shear forces. The biomimetic mechanism of the fourth joint forms corresponding slots for different diameters of rod parts to prevent disturbance caused by swinging and shaking due to shear forces. The experiment video is shown in Video S3.

### 4.4. Experimental Verification of Mobility and Assembly Functions

In actual operational scenarios, the robot cannot solely perform either mobility or assembly functions in on-orbit assembly tasks. Therefore, experimental verification combining both mobility and assembly functions was conducted.

**Figure 9:**
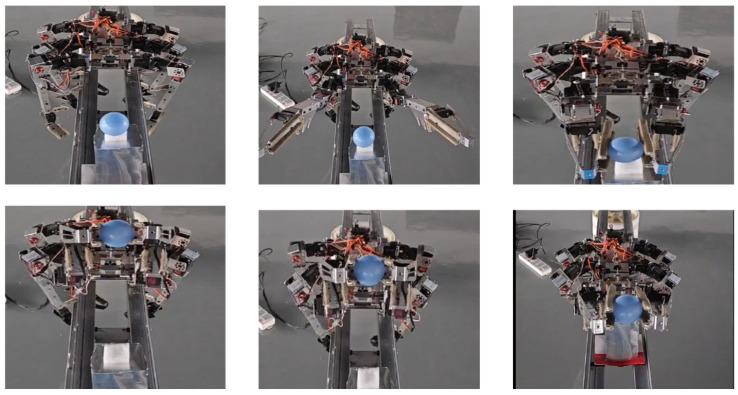
Robot carrying part movement experiment.

Figure 9 shows the combined experiment of the robot’s quadruped gait movement and gripping of spherical parts. The specific process involves the robot gripping and lifting the spherical part with its front two legs and then moving forward. As shown in the figure, using the payload platform as a reference, the robot gradually walks forward, with the position of the ball crossing over the payload platform. The experiment video is shown in Video S4.

**Figure 10:**
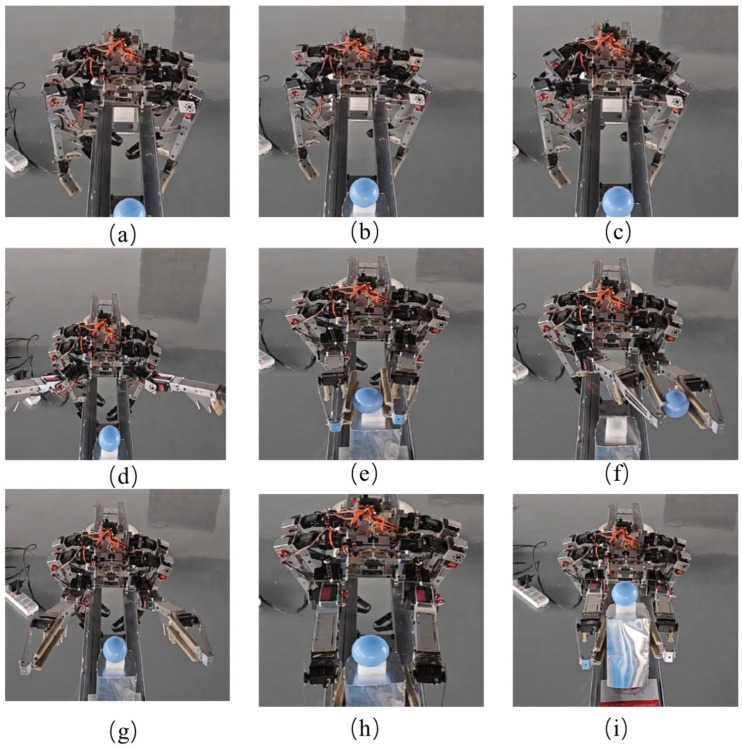
Robot movement and gripping of ball and payload platform. (**a**–**c**) The robot continuously moves forward towards the target. (**d**) It lifts the operating leg. (**e**) It grips the target ball component. (**f**) Manipulates the ball component to move. (**g**) Adjusts its posture to place the ball on the platform. (**h**) Uses the platform of the ball as the target for gripping. (**i**) Manipulates the platform while ensuring that the ball does not roll off.

Another joint verification experiment involves the robot moving from a distance to the target component and manipulating it. This places higher demands on the robot’s control. Figure 10a–c show the process of the robot moving along the truss bar. Figure 10d–f depict the operation process after the robot grips the ball. Figure 10g–i illustrate the sequence of actions where the robot sets down the ball while simultaneously gripping the platform and the ball, ensuring the ball remains on the platform without falling off. The experiment video is shown in Video S5.

## 5. Conclusions

This paper focuses on part-level in-orbit assembly of space truss structures and designs a bionic attachment robot for crawling and operating on trusses. The scheme employs a legged robot, using the tarsus of the world’s largest beetle, the Dynastes Hercules, as a bionic model. The previously researched bionic mechanism has been refined and applied to the robot, enabling flexible movement on the truss. Through an overall–local method, the kinematics and dynamics of the robot are deeply analyzed, and dynamics calculations are performed using the Kane method. The robot’s legs are divided into swinging legs, supporting legs, and assembly legs, effectively achieving both movement and assembly functions. Additionally, the robot body is designed with variable degrees of freedom to increase the working space when manipulating parts, providing the necessary prerequisites for subsequent precision assembly of truss components.

We completed experiments on the transition between six-legged and four-legged gaits on the truss, successfully solving the stability issues in robot movement and gripping. Building upon the achieved mobility on the truss, we further accomplished complex actions of carrying and manipulating parts. These experimental results indicate that this design scheme is highly feasible and has significant reference value for completing part-level in-orbit assembly tasks for space trusses. In summary, this research combines bionic design with robotics technology to develop an efficient and stable six-legged robot system, providing a novel solution for the movement and assembly of space truss structures. This study not only lays a solid theoretical and practical foundation for subsequent applications of space robots but also paves the way for the future development of bionic robotics technology.

Looking forward, we will continue to optimize the robot’s design and control algorithms, continuously enhancing its autonomy and operational precision. Further research will focus on obtaining intelligent gait planning and dynamic control schemes through reinforcement learning to improve the robot’s overall efficiency and adaptability. Additionally, we will verify and refine this system in more actual space mission scenarios, enabling it to meet the demands of more complex and variable environmental conditions. We hope that this research will promote the development of in-orbit assembly technology for space structures and play an important role in more high-tech fields, providing new opportunities for scientific exploration and engineering applications.

## Figures and Tables

**Table 1:** Classification of size levels for assembly objects in on-orbit assembly.

Assembly Object Level	Description	Corresponding Project
Spacecraft Assembly	Assembly of large functional structures is completed in orbit by mating two or more spacecraft.	MIR, ATV
Multiple Modules Assembly	Spacecraft on-orbit assembly is autonomously completed by multiple modules/segments	ETS-VII, Hubble Telescope
Module Assembly	Large structures are autonomously assembled in orbit by modules	Dragonfly
Module Expansion	Enhancement of spacecraft functionality is achieved through module replacement technology.	Phoenix
Part Manufacturing and Assembly	Parts are manufactured through material processing and autonomously assembled in orbit	SpiderFab, Arichnaut

**Table 2:** Previous research content on simulated foot attachment mechanisms.

Research Content	Description
Study of Biological Entities	Microscopic observation of biological samples, 3D physical scanning analysis
Theoretical Model Influencing Adhesion Force	Material surface parameters and contact angle as main influencing factors
Theoretical Model Influencing Adhesion Force	Tests conducted on six representative materials at different contact angles. The maximum adhesion force ranges from 4.72 N to 22.82 N
Simulation of Tarsal Mechanisms with Different Sizes	Simulation tests on 32 groups of bionic structures of different sizes, with maximum adhesion force ranging from 80 N to 13.5 N

**Table 3:** Main dimensional parameters of the robot.

Parameter	Symbol	Length/mm
Hip	L_1_	65
Tibial	L_2_	58
Knee	L_3_	305
Ankle	L_4_	135
body	B_1_ × B_2_	188 × 239

**Table 4:** The range of motion of the robot joints.

Joint Name	Min (°)	Max (°)
Hip joint	−60	60
Tibial joint	−90	90
Knee joint	−90	90
Ankle joint	−90	90
body joint	0	90

## Data Availability

The data that support the findings of this study are available from the corresponding authors upon reasonable request.

## Supplementary Materials

The following supporting information can be downloaded at: https://www.mdpi.com/article/10.3390/biomimetics9090550/s1, Video S1: movie 1; Video S2: movie 2; Video S3: movie 4; Video S4: movie 5; Video S5: movie 3.

## Nomenclature

Since certain variables appear multiple times in the article, they are summarized below for ease of reference. The same symbol with different subscripts represents corresponding different coordinate systems and will not be listed repeatedly.

$L_{i}$	The length of the link corresponding to joint i.
$\theta_{i}$	Rotation angle of joint i.
$s_{i}$	Sine value of the rotation angle of joint i.
$c_{i}$	Cosine value of the rotation angle of joint i.
R	Rotation transformation matrix between coordinate systems.
VBG	Generalized velocity of the robot body in the global coordinate system.
θ.pLi	Input velocity in the coordinate system {Li} of the *i*-th robot leg.
$C_{i}$	Contact position of the robot leg with the global coordinates.
$K_{i}$	Matrix related to the support point Ci of the *i*-th support leg.
CmsG	Contact point at the foot end of the *m*-th swinging leg.
VXαG	Velocity vector of point X at the center of mass in the global coordinate system under situation 1.
VYβG	Velocity vector of point X at the center of mass in the global coordinate system under situation 2.
ωBG	Angular velocity of point B at the center of mass of the body in the global coordinate system.
AXαG	Acceleration vector of point X at the center of mass in the global coordinate system under situation 1.
αXαG	Linear acceleration component under the acceleration vector.
εXαG	Angular acceleration component under the acceleration vector.
fXmαG	Inertial force corresponding to the center of mass X.
nXmα G	Inertial torque corresponding to the center of mass X.
FqsmLm	Driving force of the *m*-th swinging leg in the robot leg coordinate system.
$\tau_{di}^{sm}$	Driving force of the *i*-th joint of the *m*-th swinging leg.
FXrmβ*G	Inertial force of any *r*-th component of the *m*-th swinging leg.
FYmr*G	Inertial force of the *r*-th component of the *m*-th robot leg under the assembly function.
FZmr*G	Inertial force of any *r*-th component of the *m*-th support leg.
$F_{W}$	External force acting on the analysis object.
$K_{j}$	Generalized main force of the *j*-th degree of freedom.
$K_{j}^{*}$	Generalized inertial force of the *j*-th degree of freedom.
